# Advancing Mechanical Circulatory Support: From Transplant Bridging to Precision Cardiogenic Shock Therapy

**DOI:** 10.21470/1678-9741-2025-0369

**Published:** 2026-05-06

**Authors:** Alvaro Perazzo, Roberto Lorusso, Prakash P P Punjabi

**Affiliations:** 1 Transplant Unit, Instituto do Coração, Universidade de São Paulo, São Paulo, São Paulo, Brazil; 2 Cardio-Thoracic Surgery Department, Maastricht Universitair Medisch+, Hart+Vaat Centrum, Maastricht, Limburg, Netherlands; 3 Cardiovascular Research Institute Maastricht (CARIM), Maastricht University, Maastricht, Limburg, Netherlands; 4 Working Group on Cardiovascular Surgery, European Society of Cardiology, Sophia Antipolis, Provence-Alpes-Côte d'Azur, France; 5 National Heart and Lung Institute, Imperial College London. Department of Cardiothoracic Surgery, Hammersmith Hospital, Imperial College Healthcare NHS Trust, London, United Kingdom

The gold-standard treatment for refractory chronic heart failure in symptomatic patients already on optimized medical therapy is heart transplantation. In recent years, due to donor scarcity and the evolving profile of patients in profound cardiogenic shock (CS) - whether left, right, or biventricular - mechanical circulatory support (MCS) devices have gained increasing prominence in the management of critically ill patients classified as New York Heart Association (or NYHA) III or IV, and/or Society of Cardiovascular Angiography & Interventions (or SCAI) stage D/E, and/or Interagency Registry for Mechanically Assisted Circulatory Support (or INTERMACS) profiles 1 and 2. Analysis of the 2025 International Thoracic Organ Transplant (or TTX) Registry shows that after the allocation system changes introduced in 2018, heart transplantation practices have shifted in important ways. More patients are now reaching transplantation while supported by temporary MCS (tMCS), and this change has been linked to better survival outcomes - likely reflecting shorter waiting times on the list. At the same time, the reliance on durable support devices before transplantation has declined, suggesting a more efficient and timelier pathway to transplant (Figure 1)^[1^,^2]^.

**Fig. 1:**
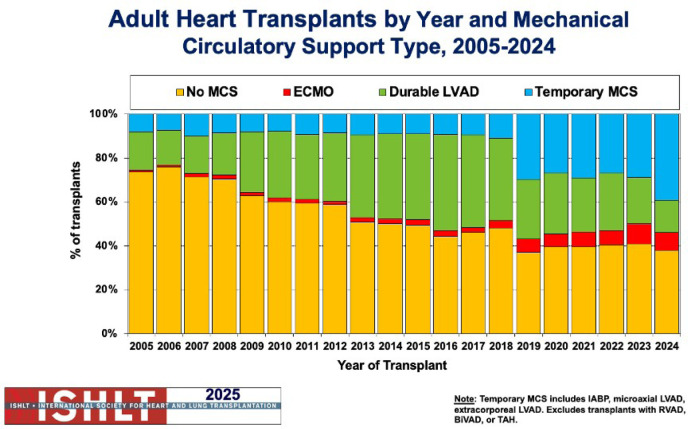
Adult Heart Transplants by Year and Mechanical Circulatory Support Type, 2005-2024. International Society for Heart and Lung Transplantation 2025. BIVAD=biventricular assist device; ECMO=extracorporeal membrane oxygenation; IABP=intra-aortic balloon pump; LVAD=left ventricular assist device; MCS=mechanical circulatory support; RVAD=right ventricular assist device; TAH=total artificial heart. Figure obtained from the registry of International Society for Heart and Lung Transplant^[11]^.

MCS can be categorized based on the required duration of hemodynamic stabilization, ranging from temporary shortand mid-term support to long-term or even to destination therapy. Among temporary devices, venoarterial extracorporeal membrane oxygenation (VA-ECMO) has emerged as a pivotal option, as it provides both left and right ventricular support in addition to gas exchange. For long-term support, the HeartMate 3 has demonstrated the most favorable outcomes in recent years and is the only currently available durable MCS in the market with long experience^[3]^. Figure 2 illustrates the main tMCS devices used in CS, highlighting their physical and hemodynamic characteristics^[4]^.

**Fig. 2:**
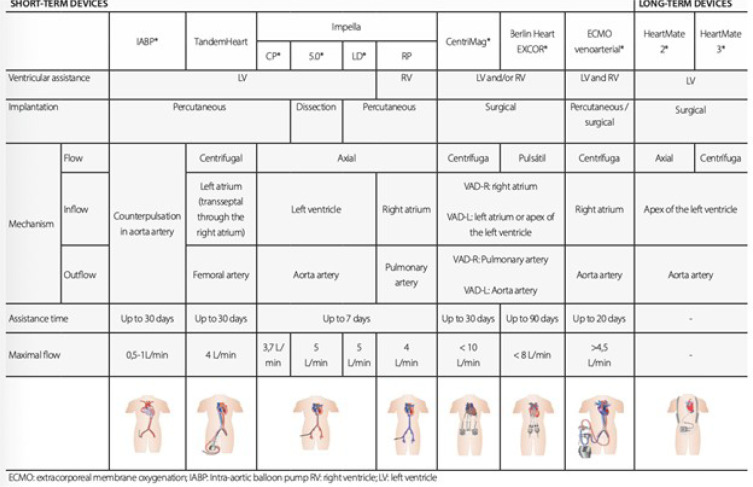
Temporary mechanical circulatory support (MCS) and durable MCS used in cardiogenic shock. ECMO=extracorporeal membrane oxygenation; IABP=intra-aortic balloon pump; LV=left ventricle; RV=right ventricle; VAD-L=ventricular assist device-left; VAD-R=ventricular assist device-right. *=. Figure obtained from Perazzo et al.^[12]^.

The intra-aortic balloon pump (IABP) remains a valuable modality for chronic heart failure, contrary to the findings of the IABP-SHOCK II Trial, a randomized clinical trial that evaluated IABP use in patients with CS following acute myocardial infarction undergoing early revascularization. Among 600 randomized patients, IABP did not significantly reduce 30-day mortality (39.7% *vs.* 41.3%; *P* = 0.69) nor improve secondary outcomes. Twelve-month follow-up confirmed no benefit in terms of mortality or quality of life. However, in cases of chronic CS refractory to medical therapy and dependent on vasoactive drugs, even a modest increase in cardiac output (0.5 to 0.8 L/min) may stabilize the patient and create a window for transplantation. In lowand middle-income countries, IABP remains an attractive and first-line strategy due to its lower cost, ease of use, and longstanding clinical integration since its first successful use by cardiac surgeon Adrian Kantrowitz in New York City in 1967^[5]^.

The Impella device, a temporary percutaneous MCS, has gained significant global traction and is increasingly employed either alone or in combination with other devices - such as ECMO - in configurations like ECPELLA (also called as ECMELLA)^[6]^. Table 1 outlines various mortality prediction scores used in patients with CS^[5]^.

**Table 1:** Selected scoring systems to predict mortality in patients with cardiogenic shock.

Score	Population	General	Neurologic	Metabolic	Hepatic	Renal	Cardiac	Hematologic	Respiratory
APACHE III	ICU	Age, temperature, chronic health score/organ failure		Lactate, pH	Bilirubin	BUN, creatinine, sodium, potassium, urine output	Cardiac arrest, heart rate, mean arterial pressure	Hematocrit, WBC	Respiratory rate, PaO₂, FiO₂
APACHE IV	ICU	Age, temperature, chronic health variables, ICU diagnosis, emergency surgery, hospitalization variables	Glasgow Coma Score	pH, glucose	Bilirubin, albumin	BUN, creatinine, sodium, urine output	Heart rate, mean arterial pressure	Hematocrit, WBC	Respiratory rate, PaO₂, FiO₂, pCO₂, mechanical ventilation
Sequential Organ Failure Assessment to predict morbidity related to sepsis	Sepsis		Glasgow Coma Score, neurological evaluation		Bilirubin	Creatinine, urine output	Mean arterial pressure or vasopressor/inotropes	Platelets	PaO₂, FiO₂
SAPS II	ICU	Age, temperature, chronic health variables, type of admission	Glasgow Coma Score		Bilirubin	BUN, sodium, potassium, bicarbonate, urine output	Heart rate, systolic blood pressure	WBC	PaO₂ if mechanical ventilation
CardShock	Cardiogenic shock	Age	Confusion	Lactate		eGFR	Ejection fraction < 40%, CAD variables		
Global Registry of Acute Coronary Events	Acute coronary syndrome	Age		Bicarbonate		Creatinine	Heart rate, systolic blood pressure, cardiac arrest, Killip class, ST segment changes, timing of cardiac enzyme elevation		
ORBI to estimate risk of development of in-hospital CS	STEMI treated with PCI without CS at admission	Age	Prior stroke	Hyperglycemia			Cardiac arrest, heart rate, systolic BP, Killip class, anterior MI, post-PCI TIMI flow < 3, LM culprit lesion, delayed PCI		
IABP-SHOCK II	AMI-CS	Age	Prior stroke	Lactate, hyperglycemia		Creatinine	TIMI flow < 3		
SAVE	VA-ECMO	Age, weight, underlying diagnoses, cause of CS					Cardiac arrest, diastolic blood pressure, pulse pressure		Duration of intubation/ventilation, peak inspiratory pressure
ENCOURAGE	VA-ECMO for AMI	Age, sex, BMI	Glasgow Coma Score	Lactate		Creatinine		Prothrombin activity	
Predict VA-ECMO	VA-ECMO			Lactate, pH		Bicarbonate			
Simple Cardiac ECMO	VA-ECMO	Postcardiotomy		Lactate		RIFLE kidney injury score			

AMI=acute myocardial infarction; APACHE=Acute Physiology and Chronic Health Evaluation; BMI=body mass index; BP=blood pressure; BUN=blood urea nitrogen; CAD=coronary artery disease; CS=cardiogenic shock; eGFR=estimated glomerular filtration rate; ENCOURAGE=prediction of cardiogenic shock outcome for acute myocardial infarction patients salvaged by VA-ECMO; IABP-SHOCK=intra-aortic balloon pump in cardiogenic shock; ICU=intensive care unit; LM=left main; MI=myocardial infarction; ORBI=Observatoire Regional Breton sur l'Infarctus; PCI=percutaneous coronary intervention; RIFLE=Risk, Injury, Failure, Loss, End Stage Kidney Disease; SAPS=Simplified Acute Physiology Score; SAVE=survival after venoarterial ECMO; STEMI=ST elevation myocardial infarction; TIMI=thrombolysis in myocardial infarction; VA-ECMO=venoarterial extracorporeal membrane oxygenation; WBC=white blood cell count.

Table adapted from Bernhardt et al.^[5]^

Establishing standardized tMCS protocols via dedicated extracorporeal life support (ECLS) teams has proven essential for institutions aiming to develop expertise in advanced support strategies. ECMO support has undergone continuous evolution, with exponential growth and increasing success rates. In addition to the conventional ECMO indications - bridge-to-recovery, bridge-to-transplant, and bridge-to-decision - five advanced clinical settings have emerged:

1. Prophylactic ECLS
2. Precision medicine: MCS and CS phenotypes
3. Protected cardiac surgery
4. ECLS for improving organ donation
5. New cannulation strategies

In the surgical setting, particularly for cardiac operations at high risk for perioperative low cardiac output syndrome, prophylactic ECLS has been proposed in select cases, such as patients with severe preoperative ventricular dysfunction or hemo-metabolic compromise, or for procedures with a high risk of hemodynamic decompensation. The rationale is to avoid emergent ECMO initiation under unfavorable conditions (*e.g.*, severe acidosis, refractory shock, multiorgan dysfunction), allowing a more controlled transition from preoperative to intraoperative and, eventually, to postoperative care. Nonetheless, such decisions must be individualized based on patient risk profile, potential ECLS-related complications (*e.g.*, bleeding, thrombosis, vascular injury), and institutional expertise as well as resources^[7]^. The ECLS-SHOCK trial, a randomized clinical trial in patients with acute myocardial infarction-related CS, demonstrated that early routine ECLS use, in conjunction with standard medical therapy and revascularization, did not reduce 30-day mortality when compared to conventional therapy alone. However, these findings do not apply to patients with advanced refractory heart failure who require MCS as a bridge-to-transplantation or destination therapy.

The integration of ECLS, particularly VA-ECMO, into the paradigm of precision medicine for CS has gained increasing interest. Precision medicine in this setting refers to the tailored selection of MCS devices based on clinical, hemodynamic, and etiologic phenotypes of CS, acknowledging the heterogeneity of this syndrome and the diverse mechanisms and therapeutic responses it entails^[3^,^8]^.

Recent evidence in post-cardiotomy ECLS suggests that a timely, proactive, and planned approach to mechanical support, rather than a reactive intervention in the face of established hemodynamic instability, may prevent severe organ hypoperfusion and improve clinical outcomes. Within this context, the concept of “protected cardiac surgery” has emerged, advocating for the early identification of high-risk patients and the planned intraoperative initiation of VA-ECMO, thereby ensuring a more stable hemodynamic transition and minimizing the need for aggressive inotropic support. Careful patient selection is essential, considering factors such as preoperative CS, biventricular dysfunction, elevated lactate levels, and advanced heart failure. In specific scenarios, such as pericardiectomy for severe constrictive pericarditis, prophylactic ECMO has proven feasible and safe to prevent right ventricular failure in the postoperative period. From a prognostic standpoint, persistent hyperlactatemia and inadequate lactate clearance during extracorporeal support are associated with worse outcomes, highlighting the importance of monitoring and correcting reversible causes of hypoperfusion. In the presence of irreversible etiologies, the prognosis remains poor^[7^,^9]^.

VA-ECMO has also gained a critical role in both extracorporeal cardiopulmonary resuscitation (eCPR) and organ preservation for transplantation, particularly in donors after brain death or circulatory determination of death (donation after circulatory death [DCD]). Whether used as vital support prior to death or as a postmortem preservation tool, ECMO has proven effective in expanding the donor pool, notably for kidney and liver transplantation, with favorable graft and recipient survival outcomes. Normothermic regional perfusion (NRP) - conducted via ECMO following circulatory death - allows for *in situ* perfusion with oxygenated blood, reducing ischemic injury and improving transplant outcomes, especially for kidneys and livers. NRP is already a standard practice in several European countries and is being implemented elsewhere with evidence of superior results compared to traditional rapid retrieval^[10]^.

Studies have demonstrated that organs retrieved from donors supported with ECMO, including those who underwent eCPR, show graft and recipient survival rates comparable to conventional donors, provided strict selection criteria are met. Advanced centers have integrated eCPR and DCD protocols, prioritizing initial resuscitation attempts and transitioning to controlled donation when unsuccessful. Nevertheless, ethical and legal concerns - such as adherence to the "dead donor rule" - require transparent protocols, clear separation of treatment and donation decisions, and stakeholder engagement to maintain public trust. Despite its potential, widespread ECMO use in organ donation faces logistical, regulatory, and cost-related challenges, necessitating further standardization and robust evidence generation^[10]^. Regarding ECLS, cannulation strategies have evolved significantly in recent years across both adult and pediatric/neonatal populations, directly impacting procedural safety, efficacy, and complication profiles. The choice of cannulation site and technique should be individualized based on clinical condition, vascular anatomy, support goals (respiratory, circulatory, or both), and potential complications. Novel techniques and devices aim to optimize support and minimize complications. Dual-lumen cannulas enable venovenous ECMO via a single venous access, facilitating patient mobilization and reducing infection and vascular complication risks. Insertion can be guided by ultrasound, fluoroscopy, or bedside portable X-ray, enhancing safety in critically ill patients. Percutaneous cannulation using a modified Seldinger technique with imaging guidance has become standard in many centers, reducing morbidity compared to open surgical access. Hybrid and dynamic ECLS strategies employing multiple cannulas or varying drainage and reinfusion sites enable adaptation to patient hemodynamic needs, particularly in right or biventricular failure. Pulmonary artery cannulation, either surgical or percutaneous, offers additional right ventricular support and facilitates cardiac drainage without left-heart access. Cannulation-related complications - such as bleeding, thrombosis, vascular injury, and limb ischemia - remain significant and influence access decisions. Image-guided cannulation and multidisciplinary team training and simulation protocols have shown to reduce complications and improve support initiation efficiency^[7]^.

In the evolving landscape of advanced heart failure and CS, temporary and durable MCSs have become an indispensable component of modern cardiovascular care. While heart transplantation remains the definitive therapy for selected patients, the increasing complexity of clinical presentations and persistent donor shortages have underscored the vital role of temporary and durable MCS. Recent advancements in precision medicine, cannulation strategies, and institutional team-based protocols have broadened the indications and improved the safety profile of tMCS in both acute and planned settings. Moreover, the integration of ECMO into perioperative care, organ donation logistics, and phenotypically driven support strategies has demonstrated its multifaceted clinical utility. Nonetheless, careful patient selection, vigilant complication management, and adherence to ethical standards remain critical to maximizing outcomes. As evidence continues to emerge and technology advances, the strategic deployment of ECLS - tailored to the individual physiological and institutional context - will be key to improving survival and quality of life in this highly vulnerable population.

## Data Availability

The authors declare that data sharing is not applicable to this article as it is an editorial and no new data were created or analyzed.
